# Association of olfactory neuropathy spectrum disorder and Wolff‐Parkinson‐White syndrome: A Report of a case

**DOI:** 10.1002/ccr3.2976

**Published:** 2020-05-25

**Authors:** Mitchell R. Gore

**Affiliations:** ^1^ Department of Otolaryngology State University of New York Upstate Medical University Syracuse New York USA

**Keywords:** ear, neurology, nose and throat

## Abstract

Olfactory neuropathy spectrum disorder is characterized by hyposmia or anosmia and hypoplastic or absent olfactory bulbs. There may be an association between olfactory neuropathy spectrum disorder and Wolff‐Parkinson‐White syndrome.

## Graphical abstract

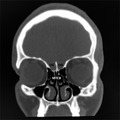

## 1 INTRODUCTION

While postinfectious, age‐related, post‐traumatic, or disease‐related (eg, diabetes, Parkinson's disease) hyposmia or anosmia is relatively common and well described, congenital hyposmia or anosmia is quite rare.^1^, ^2^, ^3^, ^4^ Kallmann syndrome is a disorder characterized by hypogonadotropic hypogonadism and congenital absence of the olfactory bulbs and is likely the most well‐known cause of congenital anosmia.^5^, ^6^ In this syndrome, patients present with lack of sexual development and delayed or absent puberty, as well as anosmia. Female patients may not undergo puberty until placed on exogenous hormones, for example, oral contraceptives. Magnetic resonance imaging (MRI) of the brain in Kallmann patients typically reveals complete absence of the olfactory bulbs. Studies indicate that a combination of mutations or gene variants may underly Kallmann syndrome. The development of the gonadotropin‐releasing hormone‐1 (GnRH) system is crucial for development of both the reproductive and olfactory/nasal placode centers in humans, and thus, mutations that affect development of the GnRH system may cause the concomitant reproductive and olfactory dysfunction seen in Kallmann syndrome patients. The olfactory placodes initially form from non‐neural ectodermal tissue bilaterally at the base of the developing vertebrate brain, and eventually develop into the neural crest tissue that forms the olfactory epithelium (OE) and olfactory ensheathing cells (OEC). Olfactory neuropathy spectrum disorder (ONSD) is a rarely seen condition in which congenital hyposmia or anosmia is associated with small or absent olfactory bulbs, analogous to the more frequent auditory neuropathy spectrum disorder (ANSD).^7^ ONSD may be present without the hypogonadotropic hypogonadism seen in Kallman syndrome, as was the case with the patient described herein. While a case of hypogonadotropic hypogonadism associated with Wolff‐Parkinson‐White syndrome (a cardiac condition associated with aberrant myocardial signal conduction that can result in tachycardia, syncope, and fatal dysrhythmias such as supraventricular tachycardia) has been reported, that patient lacked the characteristic anosmia seen in Kallmann syndrome.^8^, ^9^ In this case, we report a young patient with congenital anosmia and olfactory neuropathy spectrum disorder as well as Wolff‐Parkinson‐White syndrome.

## 2 CASE

**FIGURE 1:**
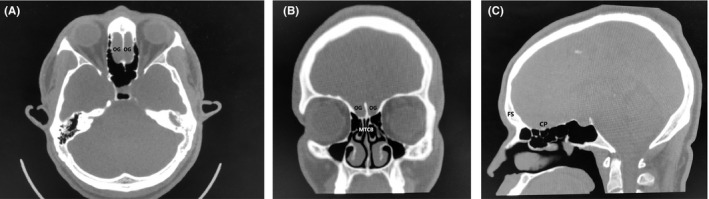
A, Axial CT view of patient with ONSD showing absent olfactory grooves (OG). B, Coronal CT view of patient with ONSD showing absent olfactory grooves (OG) and incidental middle turbinate concha bullosa (MTCB). C, Sagittal CT view of patient with ONSD showing underdeveloped cribriform plate (CP) with absent olfactory grooves and absent fenestrations for olfactory filaments and absent frontal sinuses (FS)

**FIGURE 2:**
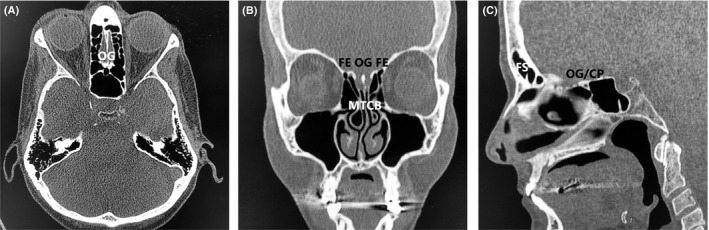
A, Axial CT view of normosmic patient showing well‐developed olfactory grooves (OG). B, Coronal CT view of normosmic patient showing normal fovea ethmoidalis (FE), well‐developed olfactory grooves (OG), and incidental middle turbinate concha bullosa (MTCB). C, Sagittal CT view of normosmic patient showing a well‐developed cribriform plate (CP) with well‐developed olfactory grooves (OG) and clearly visible fenestrations for olfactory filaments with well‐pneumatized frontal sinuses (FS)

A 27‐year‐old male immigrant originally from sub‐Saharan West Africa presented for anosmia. When questioned as to the duration of the anosmia, the patient stated that he did not think he had ever been able to smell, indicating congenital anosmia. Review of the patient's history revealed a history of Wolff‐Parkinson‐White syndrome, and three years prior to presentation the patient had undergone electrophysiologic mapping and electrocardiogram, which revealed a characteristic delta wave seen in Wolff‐Parkinson‐White and an accessory electroconduction pathway that was ablated successfully. His past history also included unspecified vision abnormalities (the patient had grossly normal vision on examination and did not wear corrective lenses), eosinophilia, and sinus headaches. Laboratory work revealed normal complete blood count (CBC) and normal thyroid‐stimulating hormone (TSH) and normal basic metabolic panel (BMP). The patient did not have a history of delayed puberty and was a never drinker and never smoker. Of note, the patient's family history was notable for a brother with Down syndrome and heart disease, and a paternal grandmother with hypertension and an unspecified arrythmia. A noncontrast maxillofacial computed tomography (CT) scan was obtained and is shown in Figure 1. Figure 1A shows an axial view showing the typical location of the olfactory groove (OG), illustrating an absence of the olfactory bulbs and the area of the olfactory groove filled with frontal lobe tissue. Figure 1B shows a coronal view of the CT showing a widened fovea ethmoidalis, absent olfactory groove with absent olfactory bulbs, and incidental bilateral middle turbinate concha bullosa (MTCB/pneumatized middle turbinates). Figure 1C shows a sagittal view, showing congenitally absent frontal sinuses (FS), and an underdeveloped cribriform plate (CP) with absent olfactory groove/olfactory bulbs and absent cribriform plate fenestrations with no outlet for olfactory filaments. In contrast, Figure 2 shows a different patient with clinically normal olfaction and normal olfactory cleft development. Figure 2A shows an axial view of the normosmic patient's CT showing a well‐developed cribriform plate and olfactory groove, with well‐developed and symmetrical olfactory bulbs bilaterally. Figure 2B shows a coronal view if the normosmic patient's CT scan, showing a well‐developed fovea ethmoidalis, well‐developed cribriform plate, and well‐visualized olfactory grooves/olfactory bulbs bilaterally. Of note, the normosmic patient has incidental bilateral middle turbinate concha bullosa. Figure 2C shows a sagittal view of the normosmic patient's CT scan, showing normally developed frontal sinuses, well‐developed and visible olfactory grooves/olfactory bulbs, and visible cribriform plate fenestrations for transmission of the olfactory filaments. The anosmic patient was counseled that the olfactory neuropathy spectrum disorder/congenital olfactory bulb absence was the cause of his congenital anosmia and was counseled on the importance of food safety (adherence to food expiration dates) and use of smoke detectors in his home. The patient declined magnetic resonance imaging (MRI) of the brain or formal olfactory testing.

## 3 DISCUSSION

Olfactory neuropathy spectrum disorder is a rare disorder characterized by hypoplastic or absent olfactory bulbs.^8^ Auditory neuropathy spectrum disorder is a more commonly seen analogue, wherein patients have varying degrees of hypoplasia of the auditory nerve and may have hearing ranging from essentially normal to profound deafness.^7^ In auditory neuropathy spectrum disorder, the otoacoustic emission test is normal since the cochlea and hair cells are typically present, but auditory brainstem response is typically abnormal given the varying hypoplasia or absence of the auditory nerve(s). In olfactory neuropathy spectrum disorder, the results of olfactory testing have not been well characterized, as ONSD is much rarer, and the formalized neurosensory testing widely published in ANSD patients is not widely published in ONSD patients. MRI can be helpful in the workup of cases of anosmia or hyposmia (congenital or new‐onset), as MRI can evaluate the presence of the olfactory nerves and whether the olfactory bulbs/olfactory nerves are normal in size. MRI can also rule out sinusitis/sinonasal polyposis or anterior skull base masses as a cause of olfactory dysfunction. GnRH testing is also helpful if hypogonadotropic hypogonadism and/or Kallman syndrome is suspected. Kallmann syndrome is the most common syndrome associated with congenital anosmia.^5^, ^6^ Kallmann syndrome is a rare disease and there is a high degree of genetic variation, and only approximately 40% of Kallmann syndrome is caused by known genetic mutations. A thorough description of the known mutations involved in Kallmann syndrome is beyond the scope of this case report, but has been well summarized and includes many gene loci, including the X‐linked KAL1 (ANOS1) (accounting for approximately 10%‐20% of Kallmann syndrome patients), FGFR1, FGF8, and many others.^5^, ^6^ The nasal placode is a source of forebrain GnRH cells, and the involvement of the GnRH pathway in both the reproductive and olfactory systems is thought to account for the common hypogonadotropic hypogonadism and anosmia seen in Kallmann syndrome. The olfactory placodes form in the ventrorostral area of the developing vertebrate brain and form from neural crest cells and non‐neural ectoderm. The olfactory ensheathing cells are also formed via this process. The epithelial cells in this area eventually form the olfactory pit and the eventual nasal cavity, and the olfactory epithelium and surrounding nasal tissue is intimately involved in the formation of these structures. There are common genes/pathways expressed in GnRH cells and OECs, and given the multiple genes implicated in Kallmann syndrome, it is likely that myriad genes may play a role in the development of the reproductive and olfactory systems that are disrupted in Kallman syndrome. Interestingly, Abs et al^8^ reported a 22‐year‐old female patient with idiopathic isolated hypogonadotropic hypogonadism and primary amenorrhoea. The patient did not fit the diagnosis of Kallmann's syndrome as she was normosmic. Of note, that patient did show facial abnormalities in the form of severe hypodontia, and the patient had an intermittent Wolff‐Parkinson‐White syndrome. They speculated that the rare phenotype suggested a nonrandom association, but given the relative rarity of both diseases, it was difficult to suggest a possible gene defect or genetic syndrome. In the present patient, the anosmia/olfactory bulb aplasia seen in Kallmann syndrome was present without the hypogonadotropic hypogonadism typically seen in Kallmann syndrome, while the present patient also had the Wolff‐Parkinson‐White syndrome seen in the patient reported by Abs et al^8^ This may simply represent a coincidental confluence of rare syndromes/disorders, or a related mutation in one or more genes involved in the pathogenesis of the various syndromes.^10^, ^11^, ^12^ Of note, human cardiomyocytes express gonadotropin‐releasing hormone receptors. Human and animal studies have shown that there are cardiac‐associated immune cells with GnRH receptors, and immunoreactive GnRH receptors have been observed in the human heart, particularly in cases of cardiac infarction.^11^, ^12^ Studies in cephalopods have suggested that GnRH may have significant activity in the cardiovascular system.^12^ The rarity of the association olfactory neuropathy spectrum disorder, Wolff‐Parkinson‐White syndrome, and hypogonadotropic hypogonadism makes potential identification of a possible common genetic mutation daunting, but genetic testing/sequencing of Kallmann and Wolff‐Parkinson‐White patients might yield candidate genes that may point to possible common genetic links and common or related protein‐protein interaction networks. Of note, Gould and Reddy reported a patient with Kallman syndrome (hypogonadotropic hypogonadism and anosmia) with second degree heart block and AV node conduction delay,^13^ further suggesting there may be an association between Kallman syndrome and GnRH‐associated cardiac abnormalities.

## 4 CONCLUSION

Olfactory neuropathy spectrum disorder is a rare syndrome characterized by hyposmia or anosmia and hypoplastic or absent olfactory bulbs. While much rarer than age‐related, postinfectious, diabetic, Parkinsonian, or traumatic causes, it should be kept in the differential, especially in younger patients with congenital or very early onset olfactory dysfunction. High‐resolution imaging through the paranasal sinuses and particularly the olfactory cleft is instrumental in diagnosis, with MRI or CT imaging preferred. Formal olfactory testing is invaluable if available. Patients should be counseled on practical issues such as food safety and smoke detection systems given the lack of olfactory sense. Genetic testing on patients with Kallmann syndrome or olfactory neuropathy spectrum disorder and/or Wolf‐Parkinson‐White syndrome may yield possible common genetic/protein pathways that may account for the rare association between olfactory neuropathy spectrum disorder and Wolff‐Parkinson‐White syndrome seen in the present patient.

## CONFLICT OF INTEREST

None declared.

## AUTHOR CONTRIBUTIONS

MRG: fulfilled the criteria and should qualify for authorship, involved in conception and design, data acquisition, drafting the manuscript, and approving the revised and final version of the manuscript. The author has managed the manuscript submission process.

## ETHICAL APPROVAL

This case report was determined to be exempt by the SUNY Upstate Institutional Review Board. The patient gave informed written consent to publish de‐identified information and clinical and radiographic images.
